# Comment on “5G mobile networks and health-a state-of-the-science review of the research into low-level RF fields above 6 GHz” by Karipidis et al.

**DOI:** 10.1038/s41370-022-00497-8

**Published:** 2022-11-24

**Authors:** Steven Weller, Murray May, Julie McCredden, Victor Leach, Dung Phung, Igor Belyaev

**Affiliations:** 1 Centre for Environmental and Population Health, School of Medicine and Dentistry, Griffith University, 170 Kessels Road, Nathan, Brisbane, QLD 4111 Australia; 2 Oceania Radiofrequency Scientific Advisory Association (ORSAA), Scarborough, QLD 4020 Australia; 3 School of Public Health, University of Queensland, St Lucia, QLD 4072 Australia; 4 Department of Radiobiology, Cancer Research Institute, Biomedical Research Center, Slovak Academy of Sciences, Bratislava, 845 05 Slovak Republic

Karipidis et al. [1] (hereinafter: Karipidis) published a scoping review investigating radiofrequency (RF) studies in the range >6 GHz, with a particular focus on the millimetre wave (MMW) band. The Karipidis review was performed against a backdrop of rising public concerns associated with the health and safety of 5th generation (5G) wireless technology [2]. Subsequently, the telecommunications industry is now using the Karipidis review to suggest “no evidence of adverse health effects from the radio waves used in 5G including mmWave” [3]. Notwithstanding the fact that no studies have investigated specific 5G frequencies and modulations, does the Karipidis review stand up to scrutiny in providing assurances of safety (no evidence of harm) that industry is suggesting? The analysis herein reveals that it does not.

A host of study design weaknesses in the existing literature were critiqued throughout the Karipidis review. In spite of the apparent lack of rigour attributed to many papers, Karipidis concluded that “experimental studies provided no confirmed evidence that low-level MMWs are associated with biological effects relevant to human health” and similarly, that radar-related epidemiological studies “presented little evidence of an association between low-level MMWs and any adverse health effects”.

This line of reasoning parallels that used previously by scientists working for the tobacco industry, whose studies repeatedly arrived at conclusions suggesting no clear determination of harm could be made [4]. This was part of a broader strategy of manufacturing doubt about the potential negative health effects of their product, as summarised by Gilbert [5]:

“The very nature of scientific exploration is to ask and answer the next question. But rather than accepting the process of scientific discovery, business interests press to have every tiny bit of uncertainty explored before any policy decision can be made, demanding proof rather than precaution—in fact, they even manufacture uncertainty. As a result, decisions are not made; policy is not advanced; problems are not addressed.”

A similar ethos is observed with the handling of scientific evidence by some governments and associated regulatory bodies in regards to radiofrequency exposures and health risks [6, 7]. The same was noted by the US Court of Appeals in the recent case against the FCC [8].

Surprisingly, the Karipidis review did not identify and discuss potential risk implications. This is of significant importance, because as Karipidis noted, the use of RF frequencies above 6 GHz is only just beginning. Best practice demands a risk management approach for the identification of all potential hazards and implementation of mitigation strategies to address these risks. This is already the case with low-dose ionizing radiation [9], but is sorely neglected for non-ionizing RF radiation [10]. Rather than waiting for harm to be established before acting, a precautionary approach to risk management is necessary [6, 11].

## Detailed analysis of Karipidis tables and selection of papers

In order to conduct an independent assessment of the Karipidis review, we performed our own literature search using the same international research libraries as Karipidis, and also accessed the Oceania Radiofrequency Scientific Advisory Association (ORSAA) database (ODEB) [12].

To perform our assessment, the Karipidis review was critiqued and classified into different categories as summarised in Tables 1 and 2. The full set of Karipidis tables, our corresponding review comments and analysis can be downloaded from the ORSAA website [13]. The results reveal issues of potential bias as well as questions around the completeness and thoroughness of the work conducted by Karipidis.

**Table 1:** Analysis summary of Karipidis et al. experimental study review (covering Tables 1–6 in their review publication).

Critique Category	Description
Incorrect Biological System	Karipidis specified “Bacteria and Yeast*”* in more than thirty experimental studies that were reviewed. This classification is not entirely appropriate because bacteria and yeast are different species and because most experiments typically expose either yeast (fungi) or bacteria, not both. A more generic description, if needed, could have been “Microbes”. However, such a level of abstraction would prevent a detailed analysis from identifying potential study replications. This is also important when it comes to potential resonance effects, as an example: DNA of yeast will have a different molecular weight compared to DNA from bacteria and so will likely respond to different but specific resonance frequencies.
Incorrect Exposure Time or Exposure Time Range	There were twenty instances where a discrepancy between the exposure duration that Karipidis indicated in their review tables and the duration specified in the reviewed papers was found.
Incorrect Frequency/ Incorrect Frequency Range/Missing Frequency	There were thirteen instances where discrepancies were identified between the exposure frequency Karipidis indicated within their tables and the exposure frequency specified in the papers they reviewed.
Incorrect Intensity/ Incorrect Intensity Range	Eighteen discrepancies were found between the exposure intensity Karipidis documented and the actual exposure intensity specified in the papers reviewed.
Misclassified/ Questionable Classification	There were seventeen instances where the inclusion of a study in a particular table in relation to biological endpoint relevance was questionable. Examples include gene expression studies being included in the genotoxicity table and vice versa.
Misstatements	There were fourteen instances where Karipidis has incorrectly stated a study finding or parameter. This is a serious issue particularly in the cases where a statistically significant finding was mis-reported as a no effect. This has direct implications for a linked study from Wood et al. [26].
Nonsensical Quality Issues	The validity of a number of quality issues raised by Karipidis can be challenged. This has direct implications to a linked study from Wood et al. [26]. Our analysis shows Karipidis has performed a quality assessment of other’s work yet their own publication suffers from serious quality deficiencies.
Findings Not Reported/ Incomplete Results	Our analysis identified forty two instances where important statistically significant biological effect findings are not disclosed by Karipidis in the results column or included in the 5G health review discussion. This has important implications for understanding biological effects that RF exposure (>6 GHz) has on biological entities and the health implications, if any, that may arise.

**Table 2:** Analysis summary of Karipidis et al. epidemiological study review (Table 7 in their review publication).

Critique Category	Description
Undisclosed Disease Risk	There were a total of eight instances identified in our analysis where epidemiological studies that identified important health risks, such as specific cancers, were not disclosed in the disease column for studies in Table 7 of the Karipidis 5G Health review.
Incorrect Case/Personnel Numbers	There were a number of instances where studies presented by Karipidis had case/personnel number discrepancies not matching actual published numbers or included case and control counts not included in the scope for review (i.e., <6 GHz).
Incorrect Odds Ratio Assignment	One instance was found where an Odds Ratio (OR) for an occupational exposure provided by Karipidis was not in the scope of review (wrong exposure type i.e., <6 GHz).
Limitations Misstatement	There were two instances where Karipidis claimed “no information on confounding factors” was found to be incorrect.
Risk Estimate Issue	Karipidis has incorrectly specified Odds Ratio in three instances where a different type of rate or ratio was used.

## Critique summary

Examination of the Karipidis 5G health review reveals many errors in classification and analysis. Some are minor, and although indicating a lack of diligence, they have no substantial implications for the outcomes identified in the papers reviewed. Of much greater concern are the number of misstatements, misclassifications, and exclusions of important findings from sound research.

The Karipidis review is at best a superficial analysis of a restricted set of available publications investigating exposures to radio frequencies in the >6 GHz range. No attempt has been made to understand or reconcile differing study outcomes. Karipidis has simply restated the results for specific endpoints, showing papers that have demonstrated statistically significant effects and those that have not. Divergent findings have been used to suggest ‘inconsistency’ as a problem, thereby diminishing the importance of biological effect findings. In contrast, our assessment [13] provides rational justifications to explain some of the divergent findings. We have also previously discussed a number of physical and biological variables, which underlie the different outcomes from studies investigating biological effects of RF exposures in general [14], and MMW exposures in particular [15]. Karipidis also attributed ‘quality’ deficiencies to a number of studies that are unjustified [13].

A literature search identified a significant number of relevant papers (at least 70 experimental papers and 16 epidemiological papers available from PubMed and ODEB) were missing from the Karipidis collection. These papers cover all major themes presented by Karipidis and more, with the majority showing statistically significant effects. By restricting the paper selection criteria, the balance of evidence can be skewed. A lack of transparency regarding papers found and ultimately discarded by Karipidis means that selection bias cannot be excluded.

Also missing from the Karipidis review is an analysis of potential publication and funding biases, which would allow the reader to assess how such influences affect study outcomes. This is often very obvious. For example, on a related topic, Carpenter [16] found that evidence for magnetic fields increasing the risk of cancer is neither inconsistent nor inconclusive (from government or independent studies), yet almost all industry supported studies fail to find any significant or even suggested associations. A similar industry funding study bias was observed with mobile phones [17].

The biased selections and assessments that have been uncovered in the Karipidis review create an unbalanced view of the science, and skew the final conclusion towards uncertainty. In contrast, when appraising all relevant findings, the evidence found in our review points to risks not fully considered by the International Commission on Non-Ionizing Radiation Protection (ICNIRP) or the Australian Radiation Protection and Nuclear Safety Agency (ARPANSA) in their respective RF guidelines and RF standards. These guidelines do not reflect the current state of scientific knowledge and are based on acute heating protection only [10], which is purely for regulatory convenience. The gulf between thermal and non-thermal evaluative frameworks has previously been discussed [6, 18].

Other important works have been omitted from the Karipidis review. Epidemiological studies suggest that RF exposures from other technologies such as radar are associated with an increased risk of hemolymphatic cancers [19], and experimental studies investigating genotoxicity in blood cells [20] have found the same. Such converging evidence requires an immediate focused investigation into RF bioeffects rather than dismissal. Other health risks potentially linked to RF exposures include pregnancy complications, fertility impairment, testicular cancer and brain cancer. These are identified in our analysis [13] and will be discussed in a future paper.

Other researchers [21] agree that the current peer reviewed science points to “predictable harm to life forms within mixed frequency mesh networks with negative consequences likely over time”. Russell assessed the literature on MMW effects on skin and eyes, the immune system, gene expression, and bacterial antibiotic resistance. Because of the shallow penetration of MMW, the skin and eyes are of significant concern. More than a decade ago, research by Feldman et al. [22] indicated that sweat ducts in the skin could behave as antennas and thus respond to MMW. The same group [23] later stated that there is enough evidence suggesting that helical sweat ducts in conjunction with wavelengths approaching the dimensions of skin layers could lead to non-thermal biological effects.

Finally, the Karipidis review lacks representation of many species, including plants, amphibians, birds, domestic animals and most importantly, insects. Therefore, readers are provided with little to no understanding of how MMWs impact these important ecological entities. This is a significant gap.

## Discussion

The above critique of the Karipidis review raises a number of ‘red-flags’. These require clarification and clear justification before telecommunications companies are given carte blanche to begin rolling out novel modulated signals to which biological systems have never been exposed.

Karipidis has conducted an investigation resulting in the exclusion of important findings, while also overemphasising quality deficiencies and inconsistencies in the data, thereby suggesting confirmation bias. Di Ciaula [24] argues that underestimating the relevance of available results (in particular those from in vitro and animal models) is ethically unacceptable, and is equivalent to saying that potential hazardous effects can only be assessed after the agent has had time to exert its harmful effects.

In this regard, Gee’s discussion [25] of risk assessment is pertinent. In “late lessons from early warnings” a variety of case studies spanning chemicals, physical agents, pathogens, and environmental issues illustrate how timing is critical for risk analysis and application of the precautionary principle. In all cases, precautionary action, or foresight based on a lower strength of evidence, would have lowered the burden of disease, reduced unnecessary suffering and prevented many premature deaths.

## Conclusion

In our opinion, the Karipidis review provides insufficient evidence of safety, which is being used by Industry [3] as justification for the planned densification and ubiquitous use of radiofrequencies >6 GHz as part of the 5G rollout. However, we concur with Karipidis that future experimental studies “should improve the experimental design” and “epidemiological research should continue to monitor long-term health effects in the population related to wireless telecommunications”.

The Karipidis review seemingly equates risk management with the need to confirm evidence of harm. The point at which harm becomes a public issue is far too late, given the size of the population being exposed without formal consent. We consider that risks to humans and the environment identified in past epidemiological studies [13], as well as unknown risks yet to be identified, warrant the application of a precautionary approach.

We find the Karipidis review to be both inadequate and incomplete, sending the wrong messages regarding safety assessment and public health.

## Data Availability

Data generated and analysed for the production of this comment article is freely available for download from the Oceania Radiofrequency Scientific Advisory (ORSAA) website at the following address: https://www.orsaa.org/5g-review-supplementary-material.html.

## References

[1] Karipidis K, Mate R, Urban D, Tinker R, Wood A (2021). 5G mobile networks and health-a state-of-the-science review of the research into low-level RF fields above 6 GHz. J Expo Sci Environ Epidemiol.

[2] Parliament of Australia. Committee Inquiry into 5G in Australia. https://www.aph.gov.au/Parliamentary_Business/Committees/House/Communications/5G/Submissions Accessed 28 September 2022.

[3] Telstra. 5G and EME research. 2021. https://www.telstra.com.au/consumer-advice/eme/5g-and-eme [Under heading “5G and EME Research” (paragraph 4)] Accessed 28 September 2022.

[4] Brandt AM (2012). Inventing conflicts of interest: a history of tobacco industry tactics. Am J Public Health.

[5] Gilbert SG (2009). Doubt is their product: how industry’s assault on science threatens your health. Environ Health Perspect.

[6] Weller S, Leach V, May M (2020). Comment on letter: “Post-normal science and the management of uncertainty in bioelectromagnetic controversies” by A.W. Wood. Bioelectromagnetics.

[7] Alster N. Captured Agency: How the Federal Communications Commission Is Dominated by the Industries It Presumably Regulates. Edmond J. Safra Center for Ethics, Harvard University, Cambridge, MA; 2015.

[8] Environmental Health Trust et al. vs Federal Communications Commission and United States of America. https://www.cadc.uscourts.gov/internet/opinions.nsf/FB976465BF00F8BD85258730004EFDF7/$file/20-1025-1910111.pdf Accessed 28 September 2022.

[9] ICRP. 2007. The 2007 Recommendations of the International Commission on Radiological Protection. ICRP Publication 103. Ann. ICRP 37 (2–4). 10.1016/j.icrp.2007.10.003 18082557

[10] Hardell L (2017). World Health Organization, radiofrequency radiation and health - a hard nut to crack (Review). Int J Oncol.

[11] Leach V, Bromwich D (2018). Why the precautionary approach is needed for non-ionising radiation devices. Radiat Prot Australas.

[12] Leach V, Weller S, Redmayne M (2018). A novel database of bio-effects from non-ionizing radiation. Rev Environ Health.

[13] ORSAA. A critical analysis of Karipidis 5G Health Review. 2022. https://www.orsaa.org/5g-review-supplementary-material.html Accessed 28 September 2022.

[14] Belyaev IY (2010). Dependence of non-thermal biological effects of microwaves on physical and biological variables: implications for reproducibility and safety standards. Eur J Oncol.

[15] Belyaev IY, Shcheglov VS, Alipov ED, Ushakov VD (2000). Nonthermal effects of extremely high-frequency microwaves on chromatin conformation in cells in vitro - Dependence on physical, physiological, and genetic factors. IEEE Trans Micro Theory Tech.

[16] Carpenter DO Extremely low frequency electromagnetic fields and cancer: How source of funding affects results. Environ Res. 2019; 178: 10.1016/j.envres.2019.108688. 10.1016/j.envres.2019.108688 31476684

[17] Huss A, Egger M, Hug K, Huwiler-Muntener K, Roosli M (2007). Source of funding and results of studies of health effects of mobile phone use: systematic review of experimental studies. Environ Health Perspect.

[18] Belyaev I Main regularities and health risks from exposure to non-thermal microwaves of mobile communication. In: 14th International Conference on Advanced Technologies, Systems and Services in Telecommunications (TELSIKS), Nis, Serbia IEEE. 2019. 111-6. 10.1109/TELSIKS46999.2019.9002324.

[19] Peleg M, Nativ O, Richter ED (2018). Radio frequency radiation-related cancer: assessing causation in the occupational/military setting. Environ Res.

[20] Zaroushani V, Khavanin A, Mortazavi SB (2014). Nonthermal effects of radar exposure on human: a review article. Iran J Health Saf Environ.

[21] Russell CL (2018). 5 G wireless telecommunications expansion: Public health and environmental implications. Environ Res.

[22] Feldman Y, Puzenko A, Ben Ishai P, Caduff A, Agranat AJ Human skin as arrays of helical antennas in the millimeter and submillimeter wave range. Phys Rev Lett. 2008; 100: 10.1103/PhysRevLett.100.128102. 10.1103/PhysRevLett.100.128102 18517913

[23] Betzalel N, Ben Ishai P, Feldman Y (2018). The human skin as a sub-THz receiver – Does 5G pose a danger to it or not?. Environ Res.

[24] Di Ciaula A (2018). Towards 5G communication systems: are there health implications?. Int J Hyg Environ Health.

[25] Gee D (2009). Late lessons from early warnings: towards realism and precaution with EMF?. Pathophysiology.

[26] Wood A, Mate R, Karipidis K (2021). Meta-analysis of in vitro and in vivo studies of the biological effects of low-level millimetre waves. J Expo Sci Environ Epidemiol.

